# Graphene-based 2D constructs for enhanced fibroblast support

**DOI:** 10.1371/journal.pone.0232670

**Published:** 2020-05-18

**Authors:** Ingrid Safina, Shawn E. Bourdo, Karrer M. Algazali, Ganesh Kannarpady, Fumiya Watanabe, Kieng Bao Vang, Alexandru S. Biris

**Affiliations:** Center for Integrative Nanotechnology Sciences, University of Arkansas at Little Rock, Little Rock, AR, United States of America; Northwestern Polytechnical University, CHINA

## Abstract

Complex skin wounds have always been a significant health and economic problem worldwide due to their elusive and sometimes poor or non-healing conditions. If not well-treated, such wounds may lead to amputation, infections, cancer, or even death. Thus, there is a need to efficiently generate multifunctional skin grafts that address a wide range of skin conditions, including non-healing wounds, and enable the regeneration of new skin tissue. Here, we propose studying pristine graphene and two of its oxygen-functionalized derivatives—high and low-oxygen graphene films—as potential substrates for skin cell proliferation and differentiation. Using BJ cells (human foreskin-derived fibroblasts) to represent basic skin cells, we show that the changes in surface properties of pristine graphene due to oxygen functionalization do not seem to statistically impact the normal proliferation and maturation of skin cells. Our results indicate that the pristine and oxidized graphenes presented relatively low cytotoxicity to BJ fibroblasts and, in fact, support their growth and bioactivity. Therefore, these graphene films could potentially be integrated into more complex skin regenerative systems to support skin regeneration. Because graphene’s surface can be relatively easily functionalized with various chemical groups, this finding presents a major opportunity for the development of various composite materials that can act as active components in regenerative applications such as skin regeneration.

## 1. Introduction

Graphene, a member of the carbon-based materials family, has rather unique physical, chemical, electrical, and mechanical properties [1,2]. These properties originate from its structure—a two-dimensional single layer sheet of carbon atoms arranged in a lattice of hexagonal rings. While the idealized bonding structure of graphene is a multitude of alternating single and double bonds (sp^2^ hybridization), the bonding is highly delocalized, creating a large amount of π-electrons within the layers of graphene [3,4]. Because of these properties, graphene has found versatile usage in a variety of areas, including electronics [5,6], energy production and storage [7,8], bio-sensing [9,10], and biomedicine [11,12].

In the biomedical field, graphene and its derivatives (two or more layers) have been intensively studied, with focus given to properties such as size, shape, and surface chemical functionalization with different functional groups, as well as its integration into various composite systems. Graphene has been used as a cell scaffold for new tissue formation [13], as well as in cell imaging, targeting, and drug delivery [14] and photothermal therapy [15]. Its surface properties are highly biocompatible, allowing cell adhesion, growth, and proliferation [16]. Despite its many successful biomedical applications, there is uncertainty surrounding the toxicity of graphene and graphene-based materials’ biodistribution in biological systems [17,18]. While some studies have reported observing cytotoxicity caused by exposure to graphene and graphene-based materials in certain biological systems, many others report no significant toxicity in other biological systems [13,16,19,20]. Notably, the study by Majeed et al. describes the importance of identifying the physicochemical properties of the graphene material in order to understand the related cellular behavior. They reported that functionalizing graphene with oxygen has a direct impact on its toxicity profile [21].

Therefore, it is crucial to study and understand the toxicity effects induced by graphene in each pertinent system associated with a biomedical application. Given graphene’s carbon-based structure, it is extremely challenging to thoroughly understand and quantify graphene’s biological distribution *in vivo* and its inherent long-term impact on organs and organisms. The most significant challenge is to accurately detect graphene and distinguish between its carbon structure and the other carbon-rich environments [18]. Spectroscopic (photothermal, photoacoustic, Raman) approaches have been developed to non-invasively study the biodistribution and pharmacokinetics of graphene *in vivo*. Furthermore, a combination of Raman and photoacoustic/photothermal spectroscopies has been used to quantify down to single cell level the uptake of graphene into various cell systems [17].

Different biological systems respond differently to the same graphene material. For example, it was found that red blood cells (non-adherent cells) and skin fibroblasts (adherent cells) responded differently when exposed to aggregated graphene sheets (GS) and dispersed graphene oxide (GO) [19]. Liao et al. observed that dispersed and smaller GO were more hemolytic than larger GO and aggregated GS. However, aggregated GS was more cytotoxic towards skin fibroblasts. This study demonstrates that the particulate state of graphene-based materials has a direct impact on their toxicity towards suspended and adherent cell lines. Interestingly, graphene has been shown to have a significant ability to alter its properties once introduced into biological systems. To exemplify, graphene interacts readily with proteins, various ions, and salts in the solutions by forming a protein corona. It can react with the reactive oxygen species and, therefore, it can potentially catalyze various biochemical reactions. As a result, graphene is a complex material that has to be characterized both before almost any biological study and after it has been introduced into the biological system, pertinent to the respective study [22,23]. Thus, any accurate conclusion on the cytotoxicity of a graphene-based material should be drawn based on a thorough, complete analytical characterization of the material, which should be integrated and presented along with the biological results.

Complex skin wounds are major health and economic problem, as indicated by their elevated prevalence worldwide and corresponding financial cost for treatment [24,25]. In the US alone, close to 6.5 million people experience chronic wounds at one point in their lifetime, and more than $25 billion are spent on the corresponding wound treatment [25]. These wounds do not heal completely with standard wound therapy in an orderly and efficient manner [24]; instead, they remain stuck in one phase of wound healing or transition from one phase to the next without fully reaching complete healing [26]. Regardless of their origin or types, these wounds show similarity in persistent infection, prolonged and excessive inflammation, tissue necrosis, and inability of local cells to respond to reparative stimuli, all of which collectively contributes to failure in the healing process [27].

Tissue engineering offers the opportunity for biomaterials such as graphene and graphene-based materials to directly address prominent issues, such as the ones mentioned above, that hinder normal wound healing and new tissue formation. These biomaterials are chosen for their unique and useful intrinsic physicochemical properties, as well as for their ability to maintain those properties when combined with other materials and bioactive molecules to make them multifunctional in tissue engineering [28,29]. In skin regeneration, graphene-based materials have been used in different setups and for different purposes. For example, graphene and graphene oxide have been previously proposed along with collagen and poly(lactic-co-glycolic acid) (PLGA) as hybrid sheets to improve human dermal fibroblast growth and proliferation; the graphene materials were chosen based on their high surface area and excellent protein adhesion properties compared to PLGA/collagen sheets alone [30]. Furthermore, due to their strong non-covalent binding with a variety of bioactive agents, graphene and GO have been used as a preconcentration platform to induce stem cell differentiation, such as mesenchymal stem cells (MSCs) [31]. Remarkably, graphene-based materials have inherent bactericidal activity on a wide range of bacteria [32–34]; however, their strong interplane interactions limit their surface area and modes of actions [35]. Therefore, functionalization and surface modifications with antibiotics, peptides, and other active molecules such as metal ions have been shown to enhance graphene’s antibacterial mechanisms [35].

Our study’s aim was to assess and understand how graphene structures with various levels of oxygen functionalization may be used as substrates for skin growth and proliferation. We used pristine graphene (PG) and two of its oxygen-functionalized derivatives, a low and a high oxygen content graphene (LOG and HOG, respectively), as primary substrates for growth and proliferation of BJ fibroblast cells, a human foreskin-derived fibroblast cell line. Our results clearly indicate that an increase in oxygen content from 2.5% in pristine graphene to 8.5% and 25% in low and high oxygen graphene, respectively, drastically altered the material’s surface properties by decreasing the surface roughness and increasing the surface energy. However, these changes in surface properties did not seem to have a short-term effect on the viability and proliferative ability of BJ fibroblasts. Therefore, our findings could serve as the foundation for more in-depth work with these specific graphene materials for regenerative applications.

## 2. Materials and methods

### 2.1. Materials

#### 2.1.1. Pristine grapheme

1–1.2-nm-thick PG (Angstron Materials, product number: N002-PDR) was used as received.

#### 2.1.2. Low-and high -oxygen graphene sample preparation

The low- and high-oxygen graphene samples were prepared according to previously reported methods [18,21,36].

#### 2.1.3. Spraying graphene solutions on well plates and glass coverslips

Dispersions of each graphene (PG, LOG, and HOG) were made at a concentration of 0.5 mg/mL and then bath-sonicated for 1 hour, followed by tip sonication for 1 hour (5sec On/Off pulse) using a Sonics VibraCell Model VCX-130 equipped with a 6-mm microtip probe. 20 mL of the dispersion were then sprayed over culture-treated 24-well plates or 15-mm-diameter glass coverslips using an air-brush technique.

### 2.2. Material characterization

#### 2.2.1. X-ray photoelectron spectrocopsy (XPS)

XPS (K-alpha, Thermo Scientific, Waltham, MA) was used to identify the elemental composition of the samples, following the procedures reported [21,36]. Briefly, the powdered samples were deposited on double-sided tape and fixed to glass substrate, then irradiated with an X-ray beam (400 μm in diameter at 36 W) from a monochromated Al Kα X-ray source with an energy of 1436.6 eV. For each sample, survey scans (0–1350 eV) at a pass energy (constant analyzer energy mode) of 200 eV and 1 eV step size were run. Thermo Avantage software was used to determine the relative abundance of oxygen and carbon based on the O1s and C1s peaks, respectively. The resulting data are presented in the supporting information.

#### 2.2.2. Atomic force microscopy (AFM)

Surface morphology of the three graphene films was examined using AFM (Bruker Dimension Icon). For each sample, AFM scans were carried out on a 25-x-25-μm area using tapping mode at 8 different locations. NanoScope Analysis (ver. 1.8) software was used to determine the surface roughness. The root mean square surface roughness, *Rq*, was determined and averaged over the 8 different locations, after each image had been smoothed to remove any artifacts.

### 2.3. Sessile drop method

EasyDrop (DSA1, Kruss Company, Germany) was used to measure water contact angle by sessile drop method. ~10-μl distilled water droplets were dispensed slowly on each sample using an automated syringe controlled by a computer. A CCD camera was used to digitally capture images of water droplets at 60 frames per second, with 780 x 580 pixels. For each sample, contact angle was measured at 3 different places, and the results were averaged.

### 2.4. Cell culture methods and reagents

BJ cells (CRL-2522, American Type Culture Collection) are skin cells derived from human foreskin. The BJ cells are commonly utilized to study skin cells [19,37]. Expansion of this cell line was conducted in Eagle’s Minimum Essential Medium culture (EMEM) (ATCC 30–2003) supplemented with 10% fetal bovine serum (ATCC 30–2020) and 1% penicillin + streptomycin. For complete cell growth, the cells were kept in an incubator at 37ºC supplied with 5% CO_2_ and 100% humidity. The cell medium was changed every 2–3 days, and regular cell passage was performed every 3–5 days depending on cell confluency inside the flask.

### 2.5. Cell viability assay: Trypan blue exclusion assay

The cell viability assay based on Trypan blue staining works to distinguish live (light) from dead cells (blue). Because the plasma membrane of dead cells disintegrates and is, thus, permeable, it uptakes the blue dye, whereas the intact plasma membrane of live cells is impermeable. In this experiment, roughly 2.5×10^5^ cells were seeded on each of the graphene films sprayed in 24-well cell culture-treated plate and in 1 ml of the serum-supplemented EMEM cell medium. Cells were allowed to grow for 2 days, then trypsinized (trypsin-EDTA (0.25%), Thermo Fisher Scientific catalog # 25200056), stained with Trypan blue dye (Thermo Fisher Scientific catalog # 15250061), and counted using a hemocytometer. The number of live cells from each treatment was recorded.

### 2.6. Cell apoptosis assay: Annexin V-FITC apoptosis detection kit

Apoptosis in cells grown on graphene films was detected using Annexin V and propidium iodide (PI); where viable cells are negative for both Annexin V and PI, early apoptotic cells are positive for Annexin V, late apoptotic cells are positive for both, and necrotic cells are only positive for PI. Annexin V (eBioscience, Cat# 11-8005-74) binds to the intracellular receptors of the plasma membrane, and PI (eBioscience, Cat# 00-6990-42) binds to cellular DNA following damage to the nuclear membrane. After the Trypan blue exclusion assay, cells were washed with the binding buffer (eBioscience, Cat# 00-0055-56). Next, they were stained with Annexin V and incubated for 30 minutes at 4ºC, away from the light. Cells were washed several times to remove excess and unbound Annexin V, then stained with PI. Data was gathered with an LRSFortessa (BD Biosciences, Franklin Lakes, NJ) at the Flow Cytometry Core of the University of Arkansas for Medical Sciences (Little Rock, AR). The flow cytometry data was analyzed using FlowJo software (TreeStar, Ashland, OR).

### 2.7. Scanning electron microscopy (SEM)

Cells were seeded on 15-mm-diameter glass coverslips coated with graphene solutions, then incubated in 1-ml cell culture medium. After 2 days of cell growth, the cell culture medium was removed, and the cells were washed several times with 1x PBS, pH 7.4 (Gibco, Cat# 10010–023), to remove any traces of cell culture medium. The cells were then fixed in 3% glutaraldehyde (EMS, Cat# 16020) in 0.2-M cacodylate buffer, pH 7.2 (EMS, Cat# 11653), for 24 hours at 4ºC. The samples were washed twice for 10 minutes each time in 0.2-M cacodylate buffer then rinsed twice in distilled water for 10 minutes each time. Next, they were post-fixed in 4% osmium tetroxide solution (EMS, Cat# 19180) for 24 hours at 4ºC. As before, they were washed twice in cacodylate buffer followed by rinsing in distilled water. The samples were then subjected to a dehydration process that involved 10-minute serial incubation in graded ethanol solution at increasing concentrations of ethanol: 30, 50, 70, 80, 95, and 100% (twice for 100%). Excess ethanol was removed by a critical point dryer. Finally, the samples were mounted with double-sided carbon tape onto aluminum cylindrical stubs and coated with ~3-nm-thick carbon layers to enhance conductivity. They were imaged by a JEOL JSM-7000F SEM with a field emission gun (JEOL-USA, Peabody, MA) at 5 kV.

### 2.8. Immunofluorescence staining of intracellular and extracellular proteins by confocal microscopy

After 2 days of growth on graphene film-coated glass coverslips, cells were fixed in 4% paraformaldehyde solution (EMS, Cat# 15710-S) for 15 minutes at room temperature. Later, they were washed twice in PBS followed by a 30-minute incubation in 2% bovine serum albumin solution (Sigma-Aldrich, Cat# A7906-50G). Then, they were incubated in CD44 antibody conjugated with APC (eBioscience, Cat# 17-0441-81) and in TE-7 antibody (Millipore Sigma, CBL 271) conjugated with Alexa Fluor 488 (Abcam, Cat# 150117). Excess, unbound antibody solution was washed away with PBS, then nuclei staining in Hoechst solution 33342 (Life technologies^™^, Cat# H3570) was conducted. Finally, the samples were washed to remove excess Hoechst solution and mounted on microscope slides for imaging.

In a separate but parallel experiment, we looked at the expression of collagen I protein by these cells after a one-week incubation on graphene films. These cells were stained with anti-collagen I (ab90395) antibody conjugated with Alexa fluor 488 by following the same procedure described above. Images were collected using ZEN Black software connected to a ZEISS LSM 880 confocal microscope with Airyscan (Digital Microscopy Core Lab, University of Arkansas for Medical Sciences, Little Rock, AR). ZEN Blue software was used to quantify immunofluorescence intensity.

### 2.9. Statistical analysis

The data was analyzed by GraphPad Prism 8.0.2. software. Statistical analysis was performed using ordinary one-way ANOVA to compare variance of the mean across different populations and with Tukey’s multiple comparison test to compare variance of the mean between each population with the control population. We also used two-way ANOVA with Dunnett’s multiple comparison test where one-way ANOVA could not be applied [38,39]. P value was considered significant when p < 0.05. All experiments were performed in triplicates with at least 3 independent repeats.

## 3. Results

### 3.1. XPS analysis reveals oxygen content in all three graphene films, with HOG having the highest oxygen amount

Our XPS analysis of the graphene samples corroborates what was reported in our previous study [21], which revealed higher oxygen content in HOG than in LOG and PG. In addition, our current graphene samples also exhibited predominantly carbon-1s and oxygen-1s photoemission peaks, at ~285 and ~533 eV, respectively, as shown by survey scans (S1 Fig). Based on the area underneath each photoemission peak, as analyzed by Avantage software, the elemental percentage composition ratios of carbon to oxygen were 97.4:2.5 in PG, 91.0:8.5 in LOG, and 74.7:25.1 in HOG. By analyzing narrow-scan XPS (not shown) data, we are able to identify different chemical states of carbon atoms present in the graphene models. Similarly to Majeed et al.’s report [21], we found the predominant peak was at 284.8 eV, which represents the C-C bond of the aliphatic or aromatic carbon sp^3^/sp^2^ atom. Due to oxygen functionalization, the C1s photoemission peak broadened towards higher binding energy, with the HOG sample displaying distinct peaks. The carbon atoms bound to oxygen, resulting in new functional groups on the HOG: C-OH, C = O, and -COOH at 286, 287, and 288.5 eV, respectively. However, PG and LOG structures showed little evidence of these C-O functional groups. Instead, they exhibited strong π-π shake-up peaks at 291 eV due to the many π-bonds present in sp^2^-hybridized systems [21].

### 3.2. Higher oxygen content decreases surface roughness

**Fig 1:**
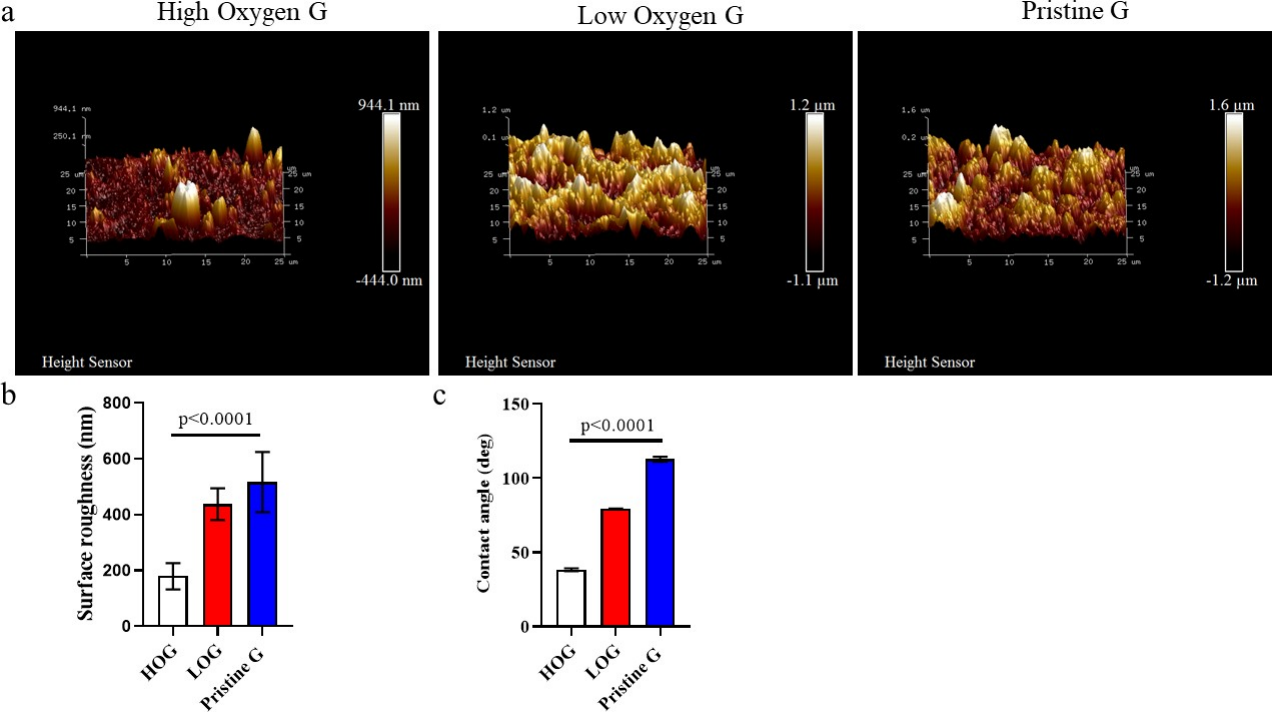
Characterization of surface roughness and hydrophilicity of high oxygen graphene (HOG), low oxygen graphene (LOG), and pristine graphene (PG). a) Representational AFM surface topography images of PG, LOG, and HOG. b) Surface roughness by NanoScope Analysis software. c) Contact angle between water droplet and surface of graphene film. Contact angle indicates a surface’s hydrophilicity: the lower the contact angle, the more hydrophilic the surface. Contact angle and surface roughness are indirectly proportional.

Representational AFM images of the three graphene films’ surface topography are shown in Fig 1A. Their surface roughness was analyzed via NanoScope Analysis software by measuring the root mean square surface roughness (Rq) at eight different locations per sample and taking their average value. The resulting surface roughness measurements are represented in Fig 1B. HOG films were found to have the lowest surface roughness, followed by low oxygen and pristine graphene, respectively (p < 0.0001). Higher oxygen content decreased the contact angle, as determined by the sessile drop method, which measures the angle formed between a water droplet and the surface of a substrate. The contact angle was greatest in PG, followed by LOG, then HOG (Fig 1C) (p < 0.0001).

### 3.3. Viability of BJ fibroblasts is maintained across all three graphene films

**Fig 2:**
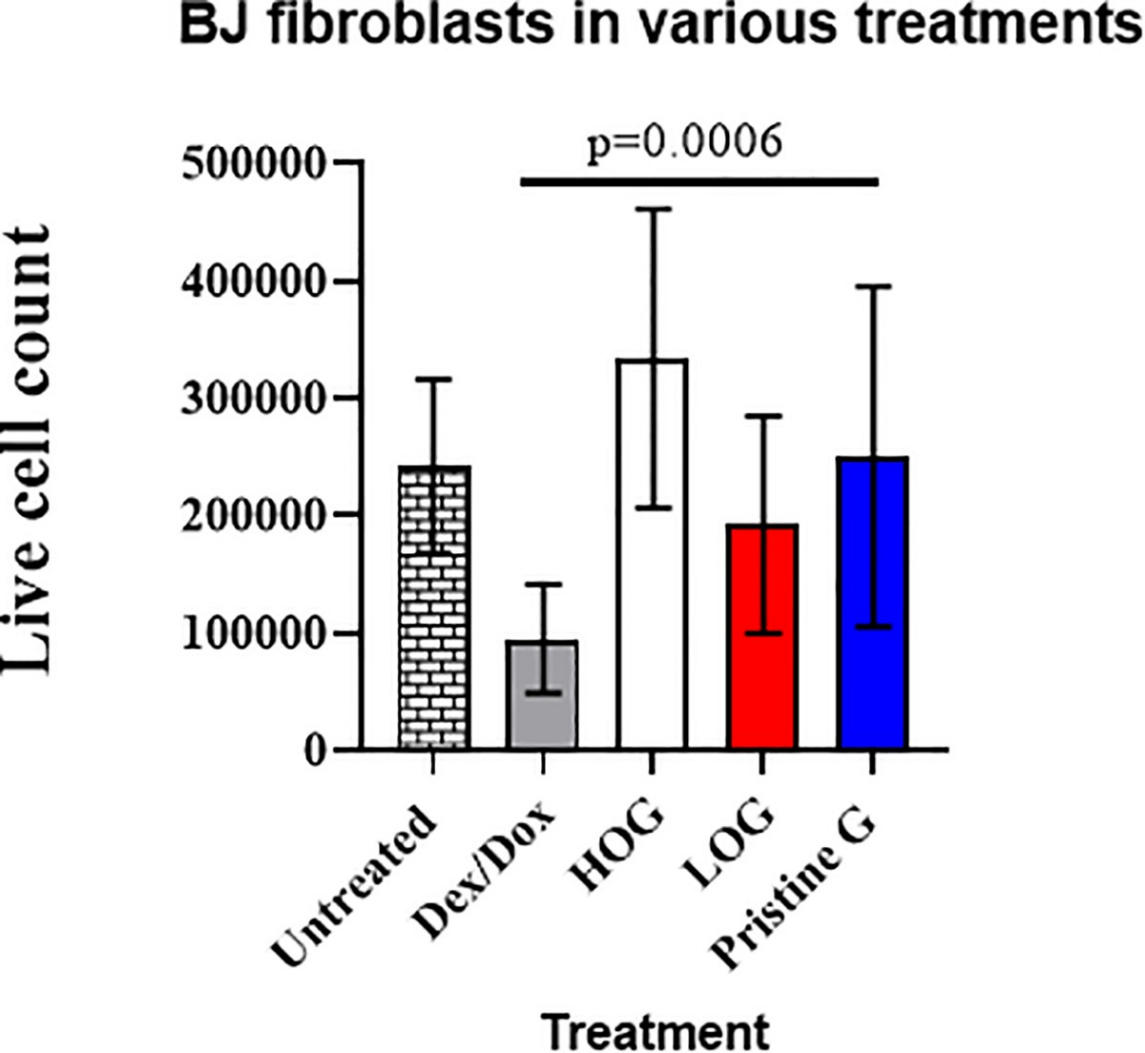
Viability assay by cell count of live cells on graphene films using Trypan blue exclusion dye. HOG has the highest live cell count, although it is not statistically different from the other two graphene films. However, there is a statistically significant difference (p = 0.006) in cell viability between the positive control (Dex./Dox.) and graphene treatments. Dex./Dox. = 400 μM dexamethasone + 5 μg/ml doxorubicin.

We assessed whether the different levels of oxygen in each of the graphene films impacted cell viability. To do so, we grew BJ fibroblasts on the graphene films for two days and then stained the cells with Trypan blue. Compared to untreated BJ cells and the positive controls (BJ cells treated with 400 μM dexamethasone (Dex.) and 5 μg/mL doxorubicin (Dox.)), HOG showed the highest viability followed by PG, then LOG (Fig 2). Notably, all three graphene films were not cytotoxic compared to the Dex./Dox. positive control (p = 0.0006).

### 3.4. Graphene films induce minimal apoptosis of BJ fibroblasts

**Fig 3:**
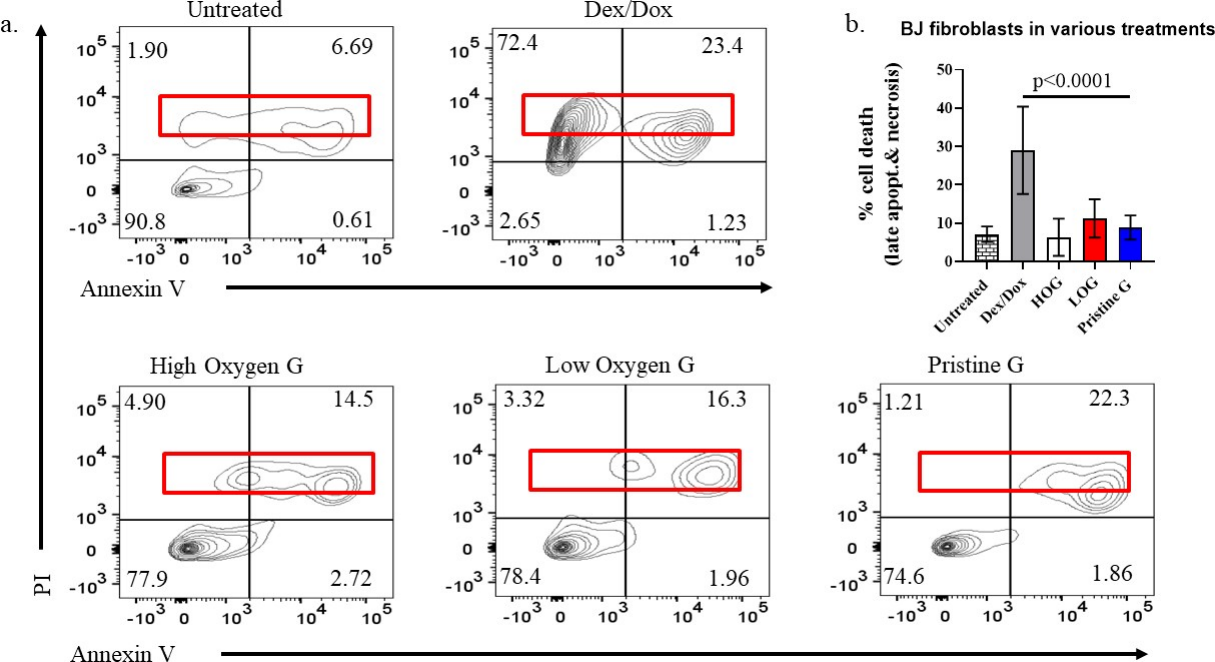
Annexin V/PI apoptosis detection assay of BJ fibroblasts on graphene films by flow cytometry. a) Representative flow cytometry graphs of apoptosis assay by Annexin V/PI staining. Cell subpopulations included in the red box represent dead cells in late apoptotic and necrotic phases. b) Compilation of all dead cells from the graphs in (a). Cell apoptosis on graphene films was compared to BJ cells untreated and treated with 400 μM Dex./5 μg/ml Dox. Our analysis indicates that graphene substrates are not cytotoxic compared to the positive control (Dex./Dox-treated) (p <0.0001).

Next, we verified the results of our viability assay by staining for cellular apoptosis. Fig 3A shows representative graphs of the Annexin V/PI assay results captured by flow cytometry for fibroblasts grown on the three graphene substrates for two days, in comparison to controls. Each graph is subdivided in four quadrants; each quadrant represents a subpopulation of cells in each viability phase (live, early apoptosis, late apoptosis, and necrosis). Lower quadrants (from left to right) represent live and early apoptotic subpopulations, whereas upper quadrants (from right to left) are late apoptotic and necrotic. Consequently, dense cellular distribution in the upper quadrants of these graphs is an indication of a potent cytotoxic agent, such as our positive control (mixture of 5 μg/ml Dox. and 400 μM Dex.). Fig 3B is a compilation of necrotic and late apoptotic subpopulations to compare the cytotoxicity of each graphene treatment with the positive and negative controls. Based on these data, HOG is the least cytotoxic; however, there is no statistically significant difference in cytotoxicity between the three graphene substrates.

### 3.5. Identification of BJ fibroblasts on graphene films by SEM and confocal microscope and differences in expression of cell surface markers on graphene compared to no graphene

**Fig 4:**
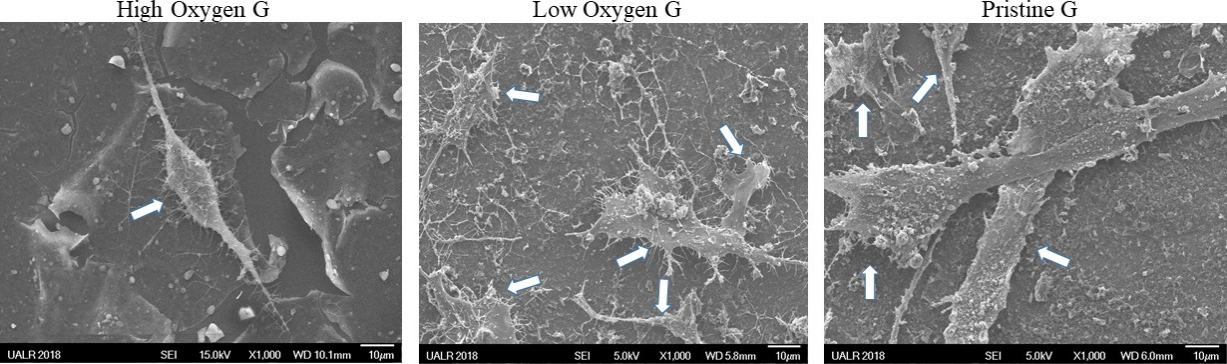
SEM depiction of BJ fibroblasts on HOG, LOG, and PG. Normal spindle-like shape of fibroblasts is maintained on all three graphene films during two days of incubation. Each white arrow points to one BJ fibroblast cell.

**Fig 5:**
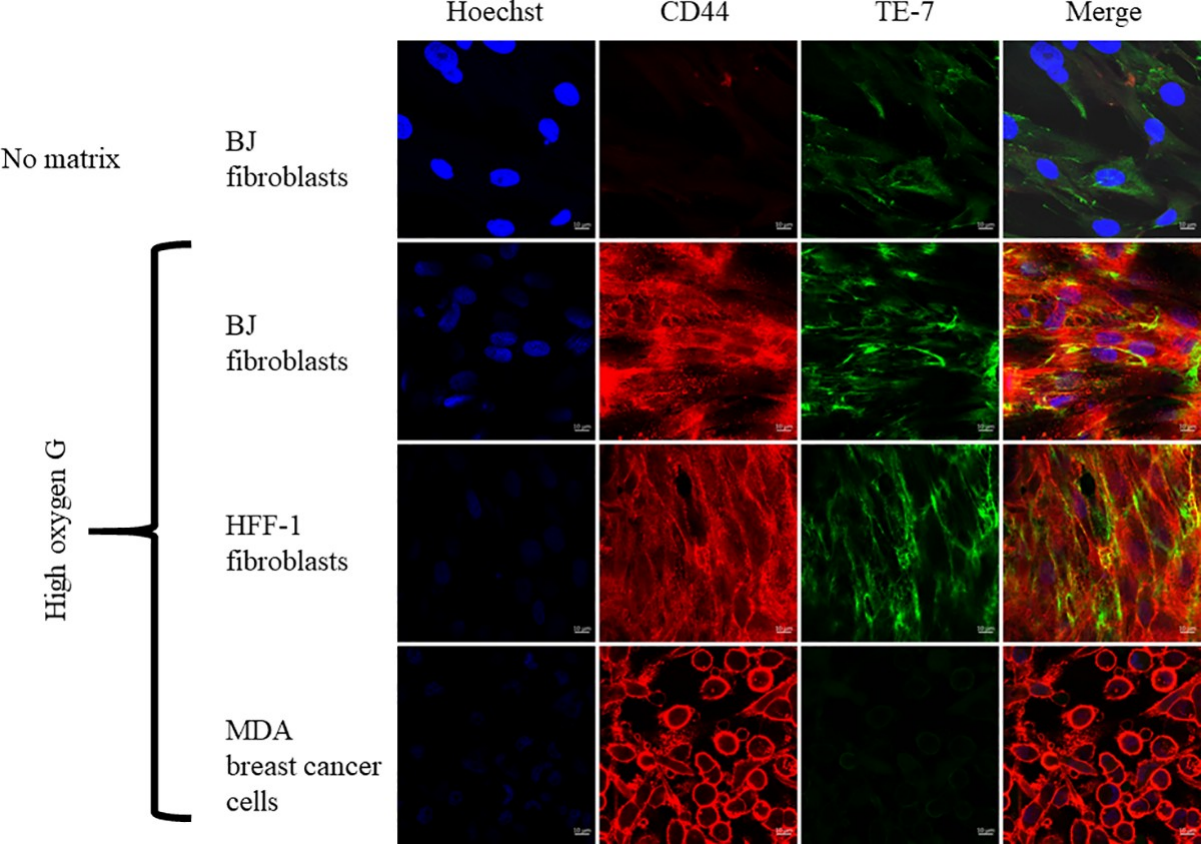
**Immunofluorescence staining of adhesion protein CD44 (red) and fibroblast marker TE-7 (green) of BJ cells on HOG film compared to no graphene (no matrix control).** Cell nuclei were stained with Hoechst dye (blue). A positive staining for CD44 is an indication of cellular adherence to and movement on the HOG. Positive stain for TE-7 indicates fibroblast cells. HFF-1 fibroblasts were used as positive control for both CD44 and TE-7; MDA cancer cells was used as negative control for TE-7 on HOG. A more intense expression of CD44 was observed on HOG than on the control (with no graphene).

Since BJ cells appeared to be viable with little to no apoptosis on graphene films, we assessed whether these cells maintained their fibroblast-like-shape as well. Using SEM, we confirmed that BJ cells did maintain their normal fibroblastic-like-shape despite going through the rigorous dehydration procedure of SEM biological sample preparation (Fig 4). To further confirm that the cells had maintained their fibroblastic nature on the graphene films, we compared the BJ fibroblasts to another fibroblastic cell line, the human foreskin fibroblasts (HFF-1), by staining for a common selective marker of fibroblasts, TE-7. In addition to TE-7, we stained for CD44, a cell surface adhesion marker to look at the cell adhesion on substrate. In addition to the HFF-1 fibroblasts used as a positive control for TE-7, we included MDA breast cancer cells as a negative control for TE-7. Both cell lines are great positive controls for CD44. BJ, HFF-1, and MDA cells were all grown on the graphene substrates for two days, then the cells were stained for TE-7 and CD44. As expected, both fibroblast cell lines BJ and HFF-1 stained positive to TE-7, with great adhesion to the substrate being observed by an intense positive staining to CD44. MDA cells also showed great adhesion to the substrate by the intense positive staining to CD44, but little to no TE-7 was expressed (Fig 5). Fig 5 shows the lower expression of CD44 and TE-7 by BJ fibroblasts on the graphene-deficient substrate (plain glass coverslip). Statistical analysis by two-way ANOVA with Dunnett’s multiple comparison test compared expression of both TE-7 and CD44 by BJ fibroblasts on and without graphene, revealing that CD44 expression was significantly higher on HOG than on the control (without HOG) (p <0.0001), but the expression of TE-7 was not significantly different (S3 Fig).

**Fig 6:**
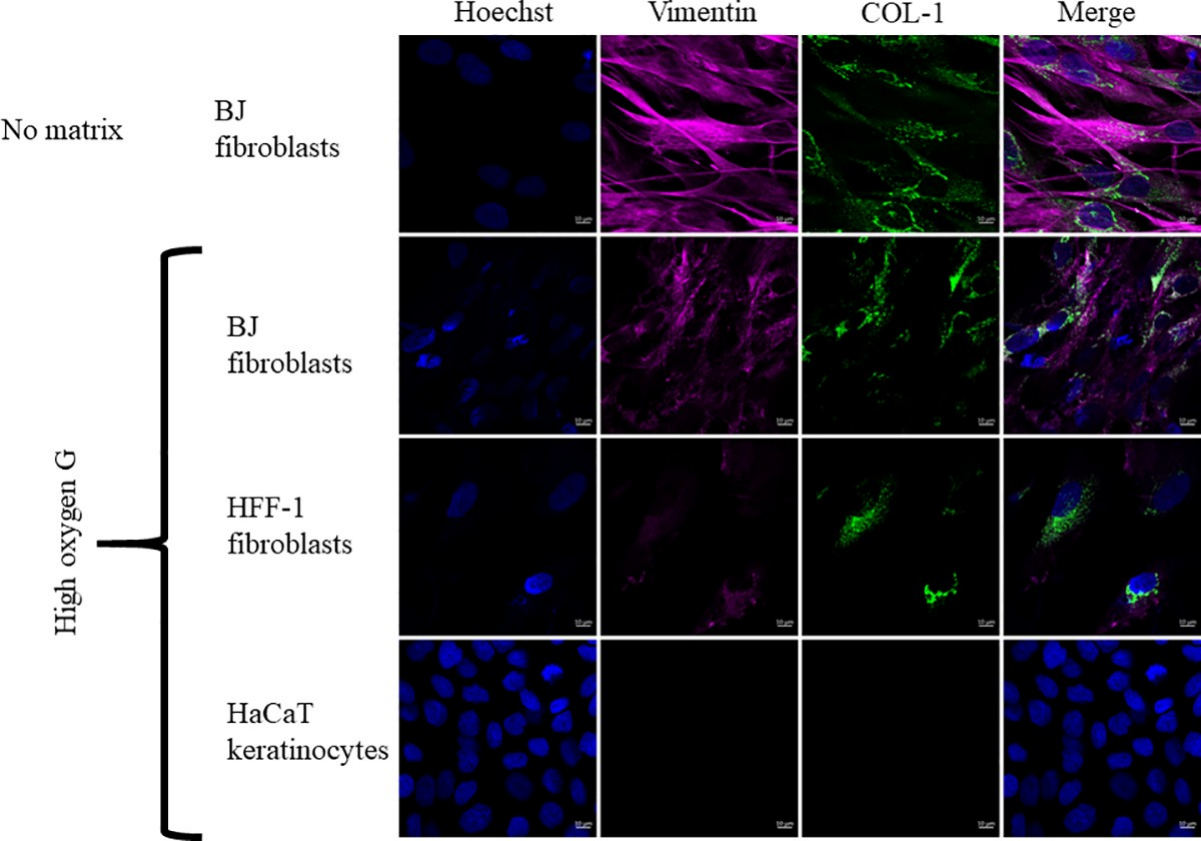
Immunofluorescence staining of collagen-1 (COL-1) by vimentin-positive fibroblasts. These images show that BJ fibroblasts are able to produce COL-1 (green) on the HOG. HFF-1 fibroblasts were used as positive control for both vimentin and COL-1, and HaCaT keratinocytes were negative control for both. COL-1 expression by BJ fibroblasts on HOG was compared to no graphene matrix (control).

Next, we examined whether BJ fibroblasts could produce collagen (COL-1), a marker of maturation, on graphene films. In this assay, we included HFF-1 fibroblasts as a positive control for COL-1 and vimentin and human keratinocytes (HaCaT) as a negative control for both markers. As expected, both BJ and HFF-1 showed a positive staining for COL-1 and vimentin on HOG, but HaCaT showed no expression of either (Fig 6). Compared to BJ fibroblasts on the HOG substrate, BJ fibroblasts on the glass coverslip without graphene (no matrix) showed significantly higher expression of both markers (S4 Fig) (p < 0.0001).

## 4. Discussion

In this study, we aimed to evaluate whether functionalizing pristine graphene with oxygen to create low and high oxygen graphene affects its use as a potential substrate for skin regeneration. We did so by assessing BJ fibroblast (a human-derived foreskin cell line) viability, morphology, and proliferation on those three graphene films. Incorporation of functional groups such as oxygen in biomaterials changes their behavior in biological systems. Importantly, these functional groups can alter a biomaterial surface’s adhesive properties towards cells [40]. Since nanosized materials are found to be taken up by cells, functional groups present on the surface of such nanomaterials are thought to influence this process, as well as their corresponding biodistribution and clearance rates [17,18]. In light of these studies, we had good reason to believe that the incorporation of oxygen groups in the graphene structure would increase its surface properties and the morphology of the resulting films. We proved that hypothesis through characterization studies of the three graphene samples (HOG, LOG, and PG).

First, using XPS, we verified the variation in oxygen content among the three graphene samples, with HOG having the highest oxygen content (~25%) compared to LOG (8.5%) and PG (2.5%) (S1 Fig). Second, the high amount of oxygen in HOG contained in functional groups C-OH, C = O, and–COOH [21,36] may have caused it to have the best dispersion in the solvent, which resulted in a more uniform spraying and, eventually, a smoother surface. This was determined by measuring surface roughness and surface energy, which was indirectly shown through contact angle measurement. In both measurements, the high oxygen content in HOG caused it to have the lowest surface roughness and contact angle (Fig 1) (p <0.0001). However, despite the differences in surface properties between HOG and the other two graphene films (LOG and PG), our data showed that BJ fibroblasts responded, grew, and proliferated similarly across the three films.

BJ fibroblasts were seeded and allowed to proliferate on these three graphene films for two days, and various assays were conducted to evaluate their growth and proliferation. First, we assessed their viability using a Trypan blue exclusion assay. From this assay, we saw an increase in cell viability on HOG in comparison to the untreated BJ cells. However, the difference in cell viability across the three graphene films was not statistically significant. Second, we analyzed graphene’s cytotoxicity using an Annexin V/PI apoptosis assay by flow cytometry. Our results showed that graphene causes very low toxicity, with HOG being the least toxic (~5%), followed by PG (<10%) and LOG (~12.5%), all of which were minimal compared to the roughly 30% toxicity of the positive control (BJ cells treated with 400 μM Dexamethasone + 5 μg/mL Doxorubicin). There was no statistical difference between the cell toxicity caused by HOG, PG, or LOG. Combined, graphene induced cell toxicity on average ~9%, significantly lower than the positive control (30%) (p <0.0001). Based on these results, HOG was used as the representative graphene substrate in the follow-up immunofluorescence studies.

Third, we assessed the graphene films’ biocompatibility with BJ fibroblasts by analyzing cell morphology and expression of two surface markers, TE-7 and CD44, after two days of incubation on HOG. From this assay, we quantified the expression of both cell surface markers on HOG and compared it to their expression in the absence of graphene (no matrix). Positive distinction of fibroblasts from other cells is challenging, in part due to a lack of specific fibroblast markers [41]. In some studies, fibroblasts have been identified by their spindle-like shape [42], positive staining to vimentin (a marker for mesenchymal cells), and negative staining to epithelial and other mesenchymal markers [43]. However, distinguishing fibroblasts based on cell shape and vimentin expression is non-specific because fibroblasts tend to acquire different shapes from tissue to tissue, and many other cells, including macrophages, may have the same spindle-like shape as fibroblasts and express vimentin [41]. Consequently, TE-7 antibody has been developed to stain the human thymic stroma in order to better understand epithelium-mesenchymal interactions during thymus development [44]. It has been found that TE-7 stains the mesodermal portion of the stroma by reacting with the fibrous tissue and vessels [44]. Goodpaster et al. [41] stained different tissues (heart and skeletal muscles, blood vessels, skin, and thymus) with TE-7 and other fibroblast-specific antibodies (1B10 and 5B5) and found that TE-7 was more specific towards fibroblasts than the other two antibodies. Additionally, in our study, we used a CD44 antibody, a common cell surface receptor involved in adhesive interactions between cells and the ECM [45], to show cell adhesion on the graphene substrates. Although CD44 is a multifunctional glycoprotein, intense expression of CD44 indicates cell migration and proliferation during cellular processes such as morphogenesis, wound healing and tissue remodeling, inflammation, and carcinogenesis [45,46]. Hence, we concomitantly utilized SEM and confocal microscopy to identify the BJs on the graphene substrates.

Using SEM, we showed that BJ fibroblasts were able to maintain their normal spindle-like shape (additional SEM images of BJs on graphene films are found in supporting information figure (S2 Fig). Using confocal microscopy, we proved BJs’ fibroblastic identity via their positive staining to TE-7 and adhesion to the substrate via positive stain to CD44. The quantitative analysis of the expression of TE-7 and CD44 on HOG in comparison to a substrate with no graphene showed a significantly higher expression of CD44 by cells grown on graphene (p <0.0001) (S3 Fig). According to this analysis, our data suggests that the graphene substrate HOG provides better cell adhesion properties than a glass coverslip substrate without graphene.

Fourth, we were able to show early matrix deposition of collagen type I by fibroblasts on graphene substrates, though it was significantly lower compared to the substrate without graphene (p <0.0001) (S4 Fig); this was analyzed after one week of incubation as an indication of their potential contribution towards artificial extracellular matrix (aECM) formation [41,47]. aECMs are particularly central in tissue engineering and regeneration, as they structurally support tissue growth and relay important mechanical and chemical signals that induce specific cellular responses, leading to new tissue growth [48,49].

Taken together, our data indicates that although CD44, a cell adhesion and migration protein, was highly expressed in BJ fibroblasts on HOG, COL-1 expression was decreased. Instead, COL-1 was more abundant in BJ fibroblasts expressing less CD44. Several studies have shown that there is a correlation between CD44 expression and extracellular matrix protein expressions, including COL-1, in fibroblasts from different tissues. However, in each of those studies, it appears that the combination of several other factors such as hyaluronanan (a significant ECM component that promotes cell-matrix interaction through CD44 receptor) and TGF-β (a potent growth factor that triggers excess deposition of collagen) in addition to CD44 expression may have played a role in increasing production of the extracellular matrix proteins, specifically COL-1 [50–52]. Therefore, while we do not negate the impact that CD44 may have on the matrix production, our results indicate the need for a more in-depth examination to understand the correlation between CD44 expression and COL-1 with a focus on skin regeneration.

Our results are in agreement with previous reports that showed pristine graphene to be a biocompatible substrate for adherent cells such as fibroblasts and osteoblasts [16,53]. Furthermore, we concur with previous studies that showed graphene surface functionalization with various chemical groups to be a major opportunity to develop various composite materials that can act as the active components in regenerative applications [30,31,34,54]. Interestingly, our work also clearly illustrates that decorating graphene with oxygen groups in various concentrations is easy to accomplish and does not seem to affect the material’s interaction with the fibroblast cells used in this study. There are many possible explanations for our results, since graphene is known to have the ability to pull various proteins (protein corona) and salts from the media, and, therefore, their surface properties will change upon exposure to the cell media. Protein corona was previously shown to have a major role in the mitigation of the toxicity of such materials [23]. Additionally, these materials have been known to scavenge reactive oxygen species and show catalytic activities [55,56].

## 5. Conclusions

We evaluated the possibility of using graphene as an active material to support cellular (fibroblast) proliferation. We investigated graphene materials with variable levels of oxygen, as induced by acid treatment, and the results showed that the oxidized graphene films had an excellent ability to support BJ fibroblast proliferation with low toxicity. Our work, therefore, clearly indicates that oxidized graphene has high potential to play a beneficial role in artificial extracellular matrices for skin regeneration.

Based on our data, we conclude that graphene and its derivatives have strong potential to benefit multiple areas of regenerative medicine, including skin regeneration. Graphitic flakes can be integrated in continuous films that support cellular proliferation and differentiation. In order to develop this graphene application, special attention needs to be focused on understanding the complex interactions between the substrates and the various cell lines, down to molecular level. It is also interesting to study the impact that graphene has on the mechanisms that control ECM-specific protein deposition and how these processes are influenced by the substrate’s properties. Another major consideration should be given to the chemistry of the graphene surface.

In summary, we have shown that altering the amount of oxygen on the surface of graphitic nanomaterials does not seem to impact their support of cellular proliferation and functions. The next step should be in-depth *in vitro* and *in vivo* mechanistic studies to further elucidate the complex interactions between graphene and various cell lines and tissues. Furthermore, the strong foundation laid by this study indicates the need for follow-on *in vivo* work to explore the potential benefits of graphene as a foundational component in skin regeneration platforms.

## Supporting information

S1 Fig X-ray photoemission spectra for graphene films. Pristine graphene (PG) (black line), low oxygen graphene (LOG) (red line), and high oxygen graphene (HOG) (blue line). The z-potential of the graphene samples with various levels of oxygen functionalizatrion has been studied and published in our earlier studies [21,57].
(TIF)

S2 Fig (TIF)

S3 Fig Histograms showing changes in immunofluorescence intensity of cell surface marker expression TE-7 and CD44 by BJ fibroblasts on HOG compared to no graphene (no matrix). a) Differences in expression of adhesion protein CD44 by TE-7-positive BJ fibroblasts on HOG compared to control (no matrix) (p <0.0001). b) HFF-1 and MDA on HOG are positive and negative controls for TE-7, respectively.
(TIF)

S4 Fig Histograms showing changes in immunofluorescence intensity of cell surface marker expression vimentin and extracellular matrix protein COL-1 by BJ fibroblasts on HOG compared to no graphene (no matrix). a) Differences in expression of extracellular matrix protein COL-1 by vimentin-positive BJ fibroblasts on HOG compared to control (no matrix) (p <0.0001). b) HFF-1 and HaCaT are positive and negative control for both COL-1 and vimentin, respectively.
(TIF)
