# Supplementary figures and images for: Graphene-based 2D constructs for enhanced fibroblast support
Source: PLoS One. 2020 May 18;15(5):e0232670. doi: 10.1371/journal.pone.0232670 (PMC7233589; doi:10.1371/journal.pone.0232670)

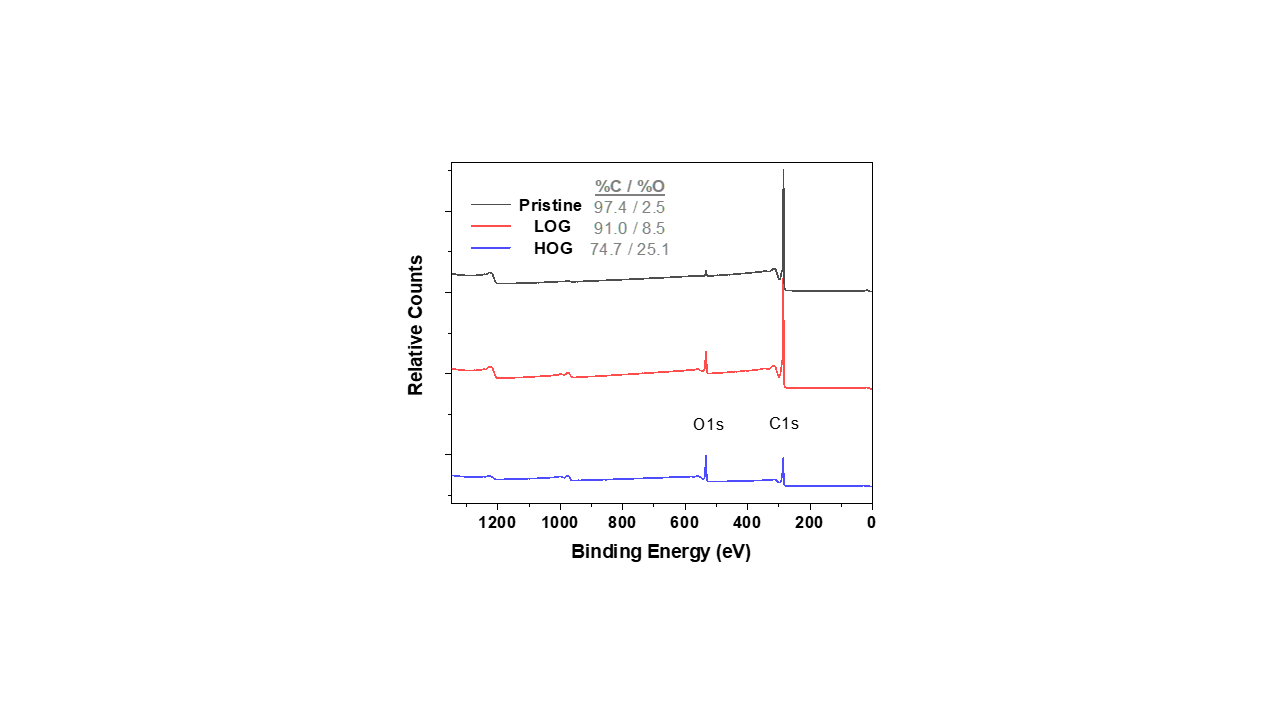

Supplement: S1 Fig — Pristine graphene (PG) (black line), low oxygen graphene (LOG) (red line), and high oxygen graphene (HOG) (blue line). The z-potential of the graphene samples with various levels of oxygen functionalizatrion has been studied and published in our earlier studies [21,57]. (TIF) [file pone.0232670.s001.tif]

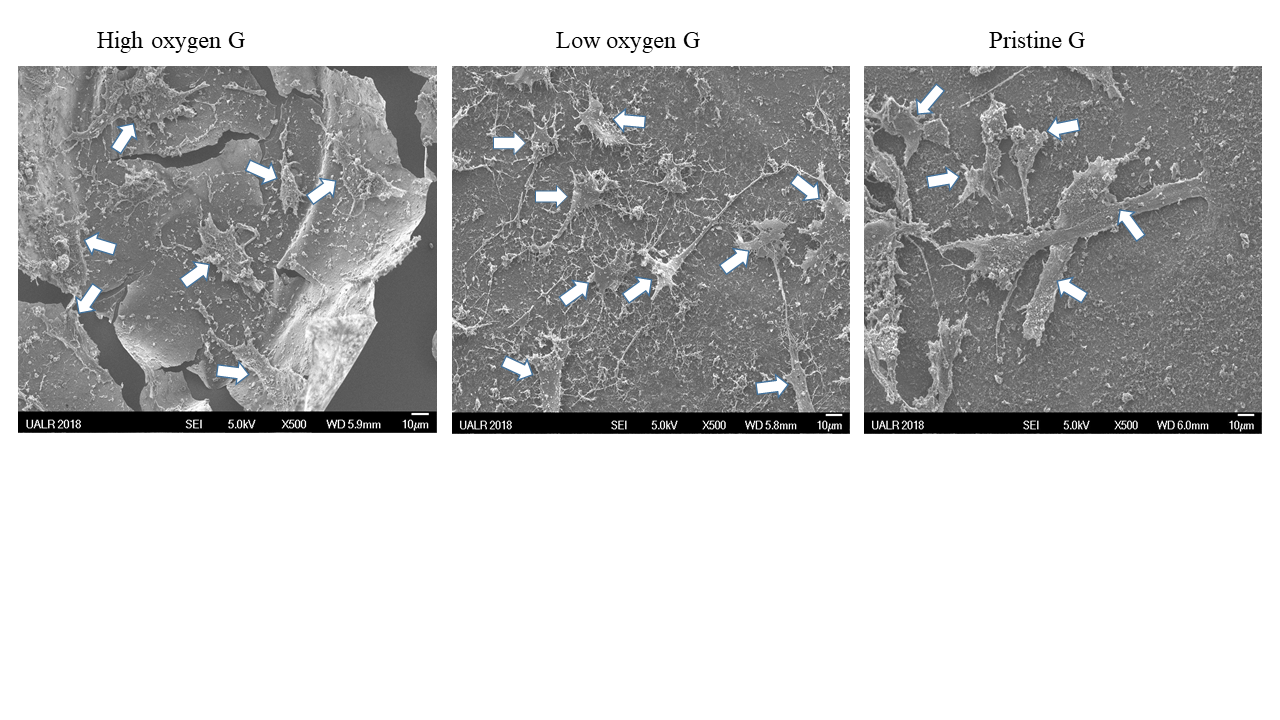

Supplement: S2 Fig — (TIF) [file pone.0232670.s002.tif]

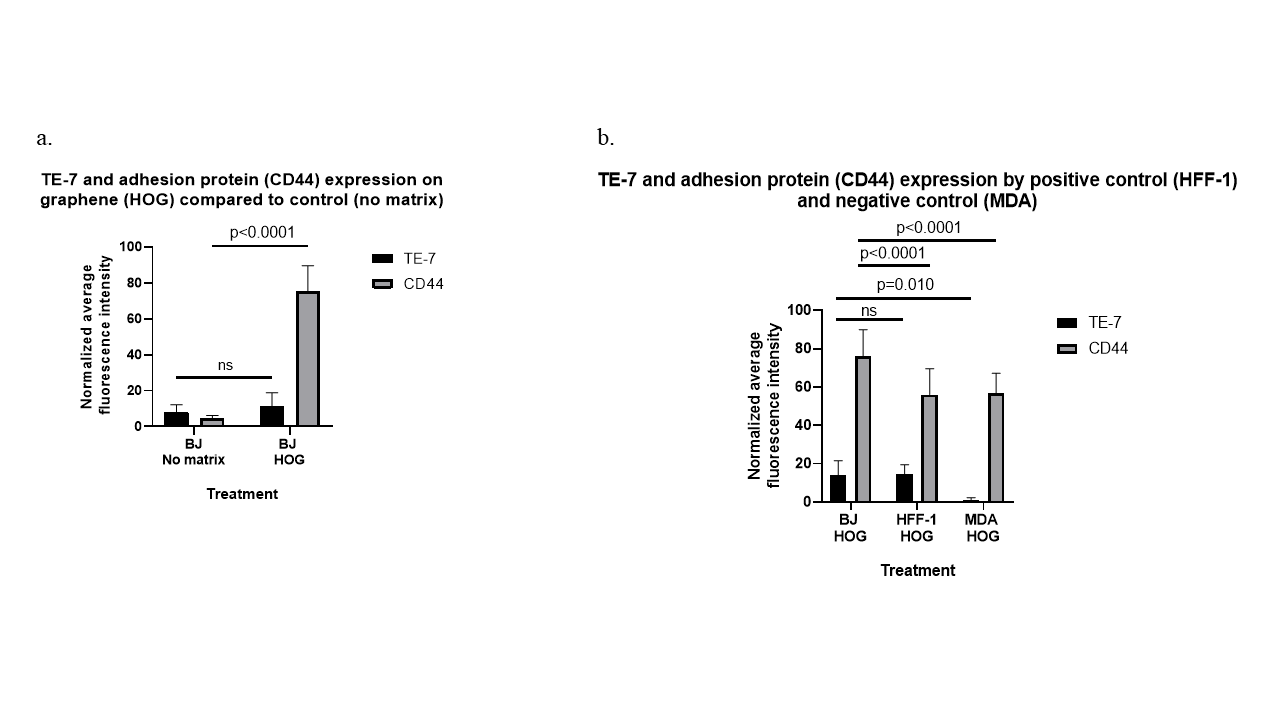

Supplement: S3 Fig — a) Differences in expression of adhesion protein CD44 by TE-7-positive BJ fibroblasts on HOG compared to control (no matrix) (p <0.0001). b) HFF-1 and MDA on HOG are positive and negative controls for TE-7, respectively. (TIF) [file pone.0232670.s003.tif]

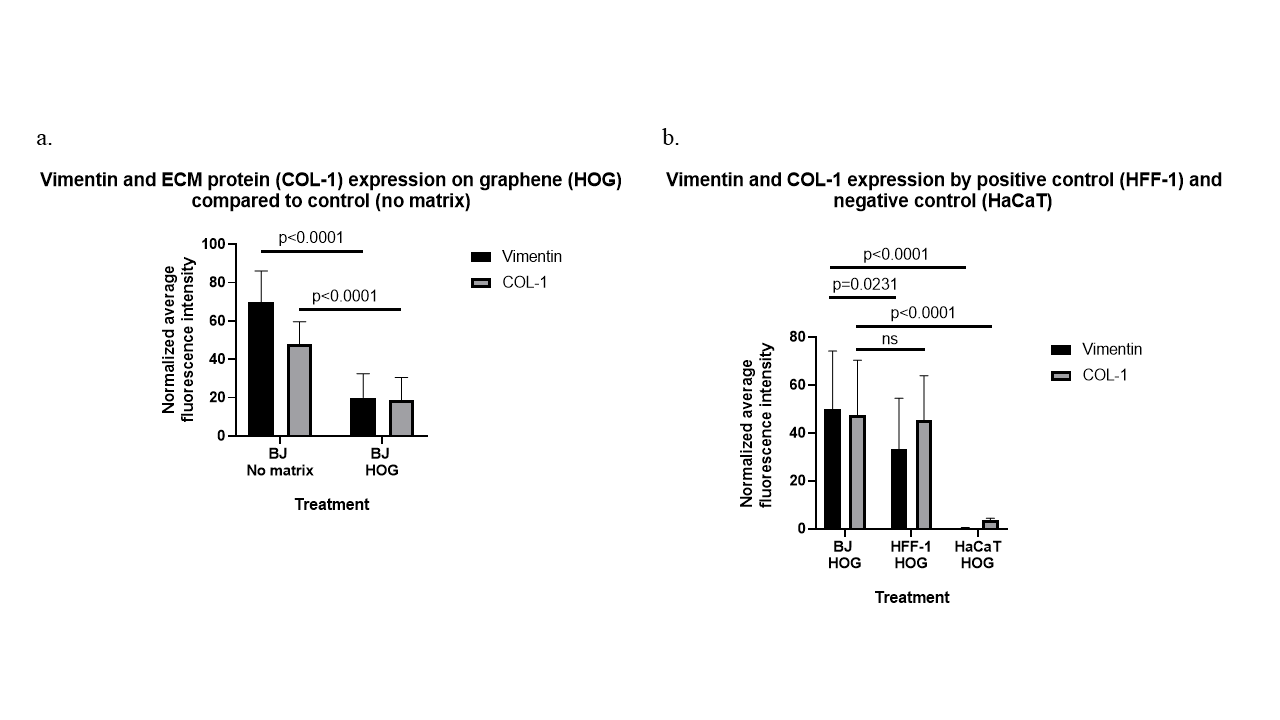

Supplement: S4 Fig — a) Differences in expression of extracellular matrix protein COL-1 by vimentin-positive BJ fibroblasts on HOG compared to control (no matrix) (p <0.0001). b) HFF-1 and HaCaT are positive and negative control for both COL-1 and vimentin, respectively. (TIF) [file pone.0232670.s004.tif]
